# Scent marking in Sunda clouded leopards (*Neofelis diardi*): novel observations close a key gap in understanding felid communication behaviours

**DOI:** 10.1038/srep35433

**Published:** 2016-10-14

**Authors:** Maximilian L. Allen, Heiko U. Wittmer, Endro Setiawan, Sarah Jaffe, Andrew J. Marshall

**Affiliations:** 1 Department of Forest and Wildlife Ecology, University of Wisconsin, 1630 Linden Drive, Madison, WI 53706, USA; 2 School of Biological Sciences, Victoria University of Wellington, PO Box 600, Wellington 6140, New Zealand; 3 Gunung Palung National Park Bureau, West Kalimantan, 78851, Indonesia; 4 Department of Anthropology, Program in the Environment, and School for Natural Resources and Environment, University of Michigan, Ann Arbor, MI, 48109, USA

## Abstract

Intraspecific communication is integral to the behavioural ecology of solitary carnivores, but observing and quantifying their communication behaviours in natural environments is difficult. Our systematic literature review found that basic information on scent marking is completely lacking for 23% of all felid species, and information on 21% of other felid species comes solely from one study of captive animals. Here we present results of the first systematic investigation of the scent marking behaviours of Sunda clouded leopards in the wild. Our observations using motion-triggered video cameras in Indonesian Borneo are novel for clouded leopards, and contrary to previous descriptions of their behaviour. We found that clouded leopards displayed 10 distinct communication behaviours, with olfaction, scraping, and cheek rubbing the most frequently recorded. We also showed that males make repeated visits to areas they previously used for marking and that multiple males advertise and receive information at the same sites, potentially enhancing our ability to document and monitor clouded leopard populations. The behaviours we recorded are remarkably similar to those described in other solitary felids, despite tremendous variation in the environments they inhabit, and close a key gap in understanding and interpreting communication behaviours of clouded leopards and other solitary felids.

Intraspecific communication is an important aspect of a species’ behavioural ecology, and the success of communication attempts can have important fitness effects for individuals. Consequently, documenting and understanding the various ways animals communicate has long been a foundation of behavioural studies^1^. Despite substantial interest in the topic, observing and quantifying communication behaviours of cryptic species in natural environments–especially carnivores that are increasingly restricted to remote and inaccessible habitats–has historically been difficult^2^,^3^,^4^. The advent of motion-triggered video cameras has greatly improved our ability to study communication in cryptic carnivores^3^,^5^,^6^, with recent results highlighting the functional role of scent marking and other communication behaviours in interactions that affect individual fitness and social organization (e.g. refs 7, 8, 9).

Solitary felids, like many mammals, rely upon scent for indirect communication and social interaction. The functional role of scent marking varies little among felids: it is predominantly used to mark and defend territories^3^,^10^ and to advertise for and select mates^7^,^8^. The presence of specific behaviours associated with scent marking, however, appears to vary considerably among felid species and lineages (Fig. 1). At present, it is difficult to ascertain whether these differences in communication are related to phylogeny, ecology or simply the lack of basic information on scent marking as data are lacking for 23% of all felid species (Fig. 1), and information on 21% of other felid species is solely from one study of captive animals (i.e. ref. 11).

Clouded leopards (Formosan clouded leopard, *Neofelis nebulosa*, in mainland Asia and Taiwan; Sunda clouded leopard, *Neofelis diardi*, in Borneo and Sumatra) are medium sized felids (males weigh up to 25 kg^12^). They are primarily nocturnal^13^,^14^,^15^,^16^, hunt a diversity of terrestrial and arboreal prey^17^,^18^,^19^,^20^, and are among the most cryptic and understudied of all felids^17^,^21^,^22^,^23^. The Bornean subspecies of the Sunda clouded leopard (*N. d. borneensis*) is classified as Endangered on the IUCN Red List, primarily due to habitat loss and fragmentation^24^. Despite some recent advances in understanding the general biology and distribution of clouded leopards (e.g. ref. 12), their social organization and forms of communication remain largely unknown. The lack of documented scent marking behaviours has led researchers to conclude that clouded leopards do not rely on scent to mark or maintain territories^17^. This, however, appears implausible given the various forms of scent marking behaviours recorded among felids, including those in the “*Panthera*” lineage (Fig. 1). Indeed, recent observations of a single clouded leopard rubbing its cheek on a scent station^25^, as well as clouded leopards often being photographed with what appears to be a flehmen response^26^, hint at the importance of scent in the species’ ecology.

Here we present results of our systematic investigation of the scent marking behaviours of Sunda clouded leopards in Gunung Palung National Park, West Kalimantan, Indonesian Borneo. We used motion-triggered video cameras to record the presence and scent marking behaviour of clouded leopards among the distinct forest types in our study area. Observations of identified individuals allowed us to determine baseline behaviour at communication sites and test a series of hypotheses about (a) spatial distribution among forest types, (b) visitation rates and fidelity to scent marking sites, and (c) differences in the frequency of observed communication behaviours (Table 1). First, due to differences among forest types in elevation, productivity and the abundance of potential prey species, we hypothesized that the occurrence of clouded leopards and their communication behaviours in the study area would differ among forest types. Second, research on other felids (e.g., pumas, *Puma concolor*^5^; snow leopards, *Panthera unica*^27^; tigers, *Panthera tigris*^3^) has shown that sites where communication behaviours are exhibited are visited repeatedly by multiple individuals. We thus hypothesized that clouded leopards would also exhibit fidelity to sites used for communication and that multiple individuals would use the same sites. Third, we hypothesized that communication behaviours would be exhibited at varying frequencies. Based on other species in the Panthera lineage (see Fig. 1), we expected the most frequently exhibited behaviours would be scraping, urine spraying and faecal marking^3^,^28^,^29^. Our observations are the first in-depth study of communication behaviours in clouded leopards, and close a key gap in our knowledge of communication behaviours among solitary felids.

## Results

### Overview

We documented 65 clouded leopard visits to 13 of the 28 (or 46.6%) monitoring sites. We documented communication behaviours at 9 monitoring sites (32.1% of all cameras, 69.2% of cameras where we recorded clouded leopards). We identified a minimum of 9 (6 males, 3 females) individual clouded leopards based on their unique spot patterns, but were unable to conclusively determine the identity of the clouded leopards in 13 of the 65 visits (20.0%). We did not document any consorting pairs of mates, but did record adult females and juveniles travelling together on 3 occasions (5.0%).

### Distribution among forest types

We documented clouded leopards in each of our seven forest types. Presence of clouded leopards generally increased with elevation, with at least 50% of cameras recording leopards in each of the three highest elevation habitats (montane, upland granite, and lowland granite) while an average of only 31% of lowland cameras recorded them (Fig. 2). The mean number of site visits by clouded leopards differed significantly among forest types (*F*_6_ = 2.61, *p* = 0.048). Mean visitation was significantly higher in lowland granite, upland granite, and montane forests than in alluvial bench, freshwater swamp, lowland sandstone, and peat swamp forests (Fig. 2).

### Fidelity to Scent Marking Sites

Among the sites visited by clouded leopards, those where communication behaviours were recorded were visited more often (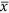 = 6.7 ± 1.2 *SE* visits) than those where they were not (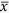 = 1.3 ± 0.3 *SE*) (*df* = 11, *t* = 2.20, *p* = 0.013). The vast majority (n = 60, 92.3%) of our clouded leopard observations were from sites where communication behaviours were recorded.

Among sites associated with communication behaviours, 7 were used by multiple individuals (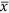 = 2.3 ± 0.3 *SE* individuals/camera, range = 1–4). Overlap appeared generally restricted to males (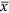 = 1.9 ± 0.3 *SE* individuals per site, range = 1–3), as we did not document any females overlapping in their use of sites.

At sites associated with communication behaviours, we documented a visit by an individual clouded leopard every 117.7 (±15.6 *SE*) days. Individuals who visited the same site multiple times did so on average every 65.7 (±6.3 *SE*, range 29.7–103.0) days.

### Communication Behaviours

We documented three distinct scent marking behaviours: scraping (at 16.7% of visits, Video 1), urine spraying (3.3% of visits, Video 2) and scat deposition (3.3% of visits) (Table 1). When scraping was exhibited, clouded leopards usually created one scrape (90% of events), but in one case an individual created two adjacent scrapes. All observations of urine spraying were associated with spraying only one object. Among scent marking behaviours, scraping was exhibited significantly more frequently than either urine spraying (*p* = 0.030) or scat deposition (*p* = 0.030) (Fig. 3).

We documented four distinct body rubbing behaviours: cheek rubbing (at 20.0% of visits, Video 3), claw marking (10.0% of visits), rolling (3.3% of visits) and tail wrapping (1.7% of visits, Video 4) (Table 1). Among body rubbing behaviours, cheek rubbing was exhibited significantly more frequent than rolling (*p* = 0.008) and tail wrapping (*p* = 0.002), but was not significantly more frequent than claw marking (*df* = 1, *X*^*2*^ = 1.63, *p* = 0.201) (Fig. 3).

We also documented vocalizations (at 3.3% of visits) (Video 6), which were among our least frequently exhibited behaviours.

We documented two investigative behaviours: olfaction (at 56.7% of visits) and flehmen response (13.3% of visits) (Table 1, Video 5). Among investigative behaviours, olfaction was exhibited significantly more frequently than flehmen response (*df* = 1, *X*^*2*^ = 22.9, *p* < 0.0001, Fig. 3).

Clouded leopards investigated the results of communication behaviours (during 58.3% of visits) more frequently than they produced cues and signals (38.3% of visits, *df* = 1, *X*^*2*^ = 4.04, *p* = 0.045). When analysed by individual behaviour, olfaction was exhibited significantly more frequently than the most frequent scent marking (scraping, *df* = 1, *X*^*2*^ = 19.0, *p* < 0.0001) and body rubbing (cheek rubbing, *df* = 1, *X*^*2*^ = 15.5, *p* < 0.0001) behaviours.

## Discussion

Results from our video recordings revealed that Sunda clouded leopards displayed 10 distinct communication behaviours. One of these behaviours–tail wrapping–has not been previously reported in any felid. Olfaction, scraping, and cheek rubbing were the most commonly recorded communication behaviours. In addition, we showed that males made repeated visits to areas they had previously used for marking and that multiple males advertised and received information at the same site (*sensu* community scrapes^5^). While our observations are novel for clouded leopards, and contrary to previous descriptions of their behaviour (e.g. ref. 17), the behaviours we recorded are remarkably similar to those described in other solitary felids, including species in the Panthera lineage (e.g. ref. 3, 4, 5 and 28, 29, 30). Our observations thus close a key gap in understanding and interpreting communication behaviours of clouded leopards and other solitary felids. Our findings are also a call for others to record behavioural observations for elusive species, especially other felids for whom such information is still missing.

**Figure 1:**
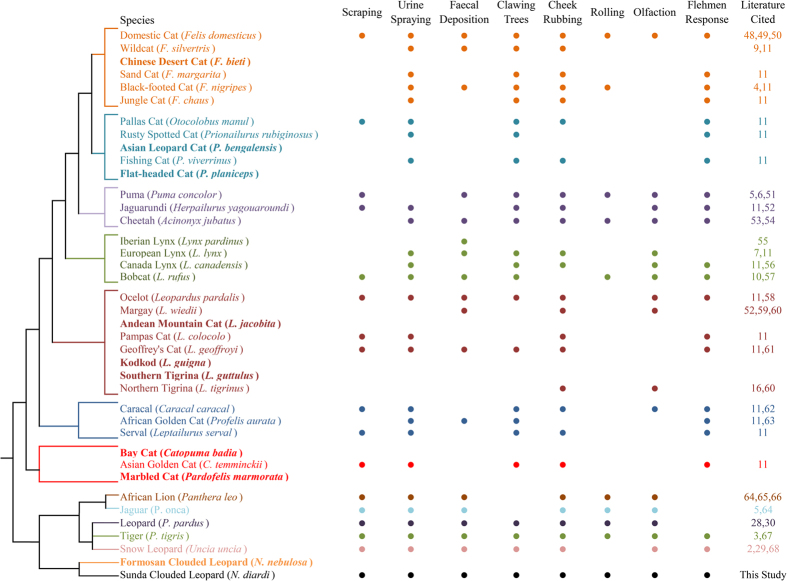
Communication behaviours reported for each felid lineage. We conducted a systematic search of peer-reviewed journals to determine the occurrence of seven different communication behaviours in all felid species. Species in bold are those for which there are no published studies on communication behaviours.

**Figure 2:**
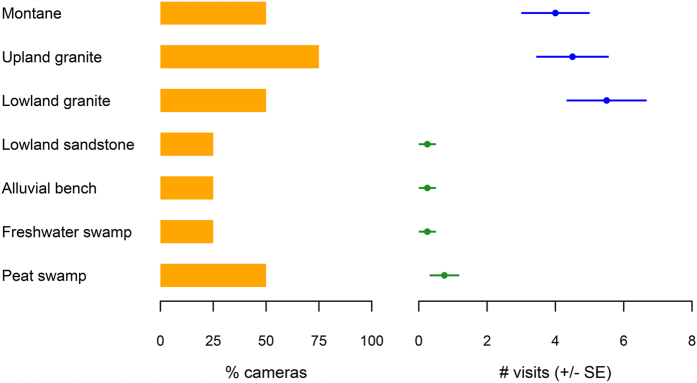


**Figure 3:**
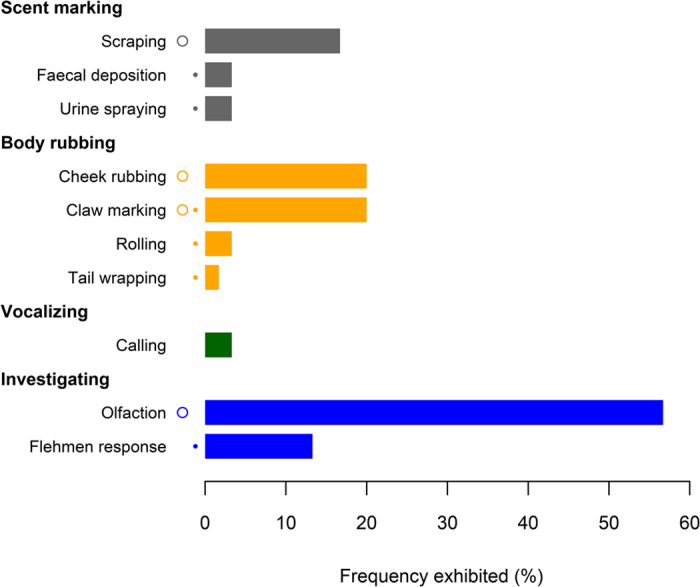
The frequency of communication behaviours observed in clouded leopards, divided into four types of behaviours. Within each group, frequencies did not differ significantly between behaviours sharing the same symbol to the left of the bars; behaviours with different symbols differed significantly.

Despite the relatively short duration of our study, we documented clouded leopards using 10 different communication behaviours (grouped into scent marking, body rubbing, vocalizing, and investigating). The use of diverse communication behaviours in clouded leopards is consistent with observations from other species of the *Panthera* lineage (Fig. 1), and overall appear most similar to leopards (*Panthera pardus*^28^,^30^) or snow leopards (e.g. ref. 4 and 29). Clouded leopards also appear similar in social organization, with males overlapping and (presumably) competing at scent marking areas, and females infrequently overlapping. Because they are similar in form and frequency to other felids, including the *Panthera* lineage, the functional ecology of clouded leopard scent marking and communication behaviours are also likely similar. Specifically, clouded leopard scent marking is likely used for intraspecific communication, with functions including both territorial marking and finding and selecting mates.

It is possible, of course, that clouded leopards also exhibit unique forms of communication, as suggested by previous studies (e.g. ref. 17), and this may include unique forms and functions of scent marking. For example, we documented tail wrapping, where the clouded leopard wrapped its tail around a tree trunk, once during the sampling period–although ongoing camera sampling in our study area has since led to at least one additional observation of this behaviour. Tail wrapping does not appear to have been previously described in literature for any other felid species, and may be unique to clouded leopards. Clouded leopards are known for their arboreal behaviours, including hanging from tree limbs from their claws and using their notably long tails for balance^22^. The wrapping of their tails around objects on trails may be related to this, or it may be used intentionally to leave scent, as with other forms of body rubbing.

Clouded leopards are among the most cryptic and understudied of all felids, and what little information is available is often anecdotal^22^,^23^,^31^. To date, only two telemetry studies have been conducted^32^,^33^, highlighting significant knowledge gaps regarding population abundances and spatial organisation (e.g. ref. 21). Our discovery of clouded leopard scent marking, their fidelity to identified scent marking areas and the use of the same scent marking areas by different males has the potential to help overcome existing limitations studying the ecology of clouded leopards. Specifically, repeated visits of identifiable individuals to scent marking areas should help improve accuracy of population estimates using non-invasive remote cameras^34^. The fidelity of clouded leopards to scent marking areas may also be used to increase the success of capturing clouded leopards and fitting them with telemetry collars. Such studies are urgently needed to develop effective conservation strategies for this endangered carnivore in Borneo.

While the variation we observed in clouded leopard abundance among the seven forest types and along the elevational gradient in our study site is intriguing, data are too few at present to warrant firm conclusions. As in other species, habitat use by clouded leopards is likely a trade-off between maximizing short and long-term access to forage and mates while simultaneously minimizing exposure to possible threats^35^. While forest types in our study area are known to differ in terms of productivity^36^,^37^, the link between variation in productivity and prey availability or other resources for clouded leopards remains unknown. Determining possible differences in prey availability among forest types, together with detailed location data from individual clouded leopards fitted with telemetry devices, would be required to understand forest type use and selection patterns in the study area, including the selection of sites for communication.

Our results highlight the remarkable similarities in communication behaviours among felids in the *Panthera* lineage, despite tremendous variation in the environments they inhabit. Because basic information on communication and scent marking is still missing for many species, we suggest that future research should now focus on understanding individual-, sex- and age-specific differences in the form or function of scent marking for felid species. This will require extensive field efforts and studies over longer time periods due to known seasonal differences in scent marking^3^,^8^,^38^. Species-specific results, such as those we have presented, are necessary to understand the evolution and ecology of intraspecific communication in felids. Many felids are rare and threatened, and the findings provided from such studies will inform the ecology and conservation of felids on both local and global scales.

## Materials and Methods

### Study Area

**Figure 4:**
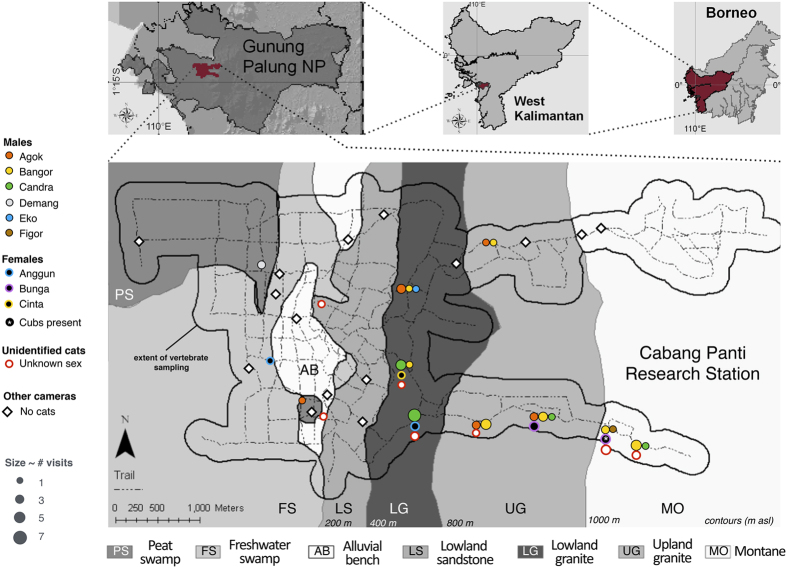
Our study area in Gunung Palung National Park in West Kalimantan, Borneo. The figure was created with Keynote 6.2.2/S 8.3 (top three panels; https://web.archive.org/web/20030207075958/http://www.esri.com/software/arcgis/index.html) and a trail map created in ArcGIS in 2010, version unknown (bottom panel; www.arcgis.com). The cameras and locations of individual clouded leopards are noted.

We conducted our study in Gunung Palung National Park, West Kalimantan, Indonesia (Fig. 4), a 1,084 km^2^ area comprising a diversity of forest types from coastal mangroves to montane forests^39^. We gathered all data in a well-established trail system around the Cabang Panti Research Station (1°13′S, 110°7′E^37^). Our research trails traverse elevations from 5 to 1119 m asl and seven contiguous forest types (in order of increasing elevation): peat swamp, freshwater swamp, alluvial bench, lowland sandstone, lowland granite, upland granite, and montane^40^,^41^,^42^. Forest types differ in soil type, drainage, floristic composition, plant productivity, and mammal densities^36^,^37^,^41^,^42^,^43^, and likely the abundance of potential prey species for clouded leopards.

### Field Methods

We placed motion-triggered video cameras (Bushnell TrophyCam, Overland Park, KS) at 28 locations to monitor wildlife from June 2015 to February 2016 (n = 5,419 trap nights). We placed four cameras in each of the seven forest types (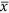 = 193.5 ± 5.6 *SE* days per camera, range 114–232), along established monitoring trails at locations we thought would increase our probability to encounter wildlife, including clouded leopards (e.g., trail intersections and ridge lines). We set the cameras to record a 20 s video every time motion was detected, with a 10 s refractory period before becoming active again. The data in this study were collected through non-invasive methods, and no animals were handled, drugged, or harmed. The research was conducted in accordance with the policies of the University of Michigan IACUC.

**Figure 5:**
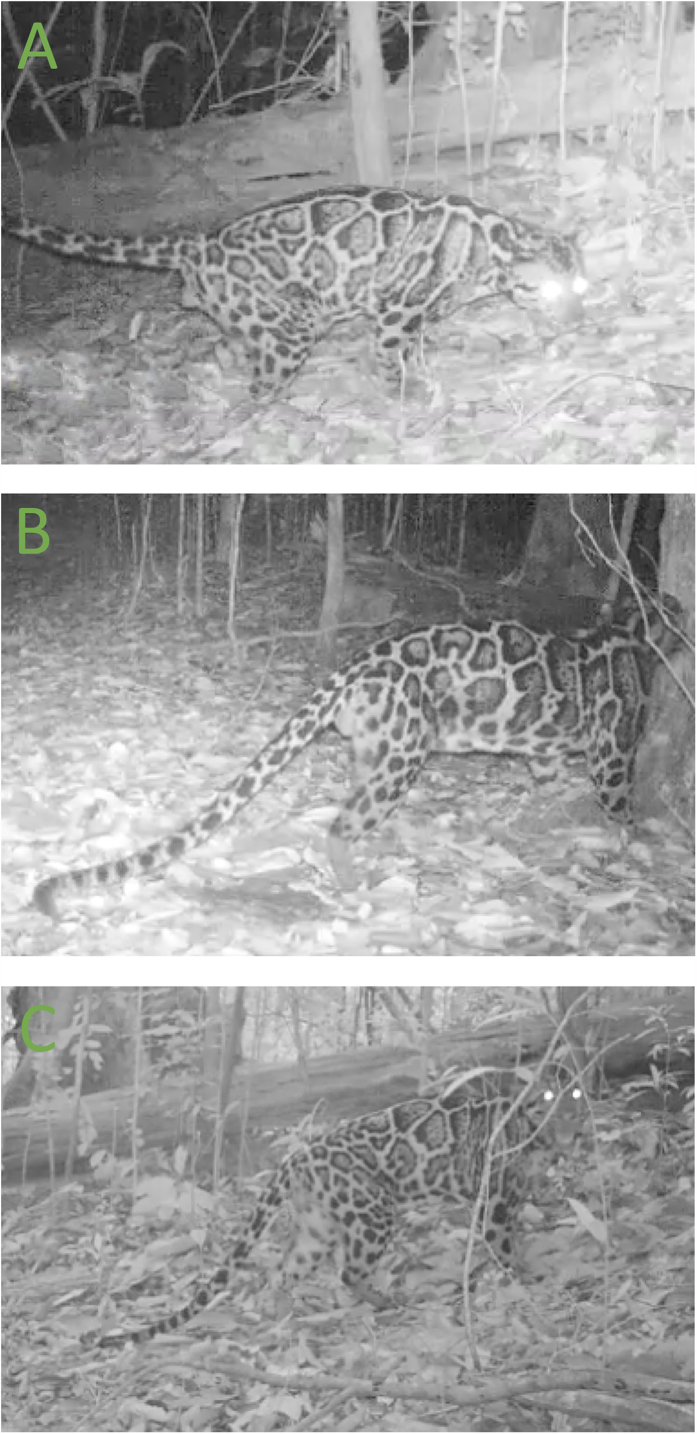
An example of how individual clouded leopards can be distinguished based on their unique spot patterns. The top and bottom photographs show the same male, while the middle photograph shows a different individual male.

We watched all video recordings to identify those that included clouded leopards. We categorized clouded leopards in these videos as male or female (based on genitalia) and noted whether they were mature or immature (immature individuals were identified based on their association with an adult female). When possible, we identified individuals using their unique spot patterns (Fig. 5). We also recorded all communication behaviours displayed by clouded leopards during each visit, including scent markings, body rubbings, investigative behaviours and vocalizations.

### Data Analyses

We used program R version 3.2.2^44^, and set alpha at 0.05 for all statistical analyses. We first calculated summary statistics; this included calculating visitation rates (as a ratio estimator^45^) by dividing visits for a given individual by the number of days a site was monitored by a camera. We then examined spatial and temporal patterns in clouded leopard distribution and communication behaviours, including their (a) spatial distribution and visitation among forest types, (b) fidelity (repeated visits) to specific localities, and (c) relative frequencies of exhibition of different behaviours.

To test if the visitation of clouded leopards varied among the seven forest types, we used a generalized linear model with a Poisson link, with the number of visits as the dependent variable and the forest type as the independent variable.

To quantify if repeated use of distinct sites was occurring, we compared visitation rates of sites with and without observed communication behaviours using a two-tailed t-test^46^. We determined the mean and range of the number of individuals using the same site, and the time interval (i.e. visitation rate) between consecutive visits.

To test if clouded leopards varied in their display of different communication behaviours, we used a series of chi-square tests^46^. For rare behaviours (n < 5 occurrences), we instead used a Fisher’s exact test^46^; in these cases, we report only the *p* values. In these analyses we included only the visits to sites where we had documented communication behaviours. We then tested if clouded leopards varied in their exhibition of producing (scent marking and body rubbing) behaviours and investigative behaviours using a chi-square test.

We performed systematic searches using Web of Science databases to review the literature on communication behaviours for each felid lineage^47^ (Fig. 1). We searched for each species (using the common name followed by the scientific name) with the combination of keywords: “scent”, “scrape”, “communication”, “sniff”, “olfaction”, “rolling”, and “claw”. We then read any publication from peer-reviewed journals published in English (while excluding obvious mismatches), and noted any documented communication and scent marking behaviours. In some cases (<5%, and never for species for which we report no publications) we were not able to obtain publications from obscure or out-of-print journals. For Fig. 1 we added the publication with the most documented communication behaviours, and then any other publications that added at least one other communication behaviour for the species^2^,^3^,^4^,^5^,^6^,^7^,^9^,^10^,^11^,^48^,^49^,^50^,^51^,^52^,^53^,^54^,^55^,^56^,^57^,^58^,^59^,^60^,^61^,^62^,^63^,^64^,^65^,^66^,^67^,^68^.

## Additional Information

**How to cite this article**: Allen, M. L. *et al.* Scent marking in Sunda clouded leopards (*Neofelis diardi*): novel observations close a key gap in understanding felid communication behaviours. *Sci. Rep.* **6**, 35433; doi: 10.1038/srep35433 (2016).

## Supplementary Material

Supplementary Information

Supplementary Video 1

Supplementary Video 2

Supplementary Video 3

Supplementary Video 4

Supplementary Video 5

Supplementary Video 6

## Figures and Tables

**Table 1:** Descriptions of the nine scent marking, body rubbing, and investigative behaviours.

Type	Behaviour	Definition
Scent Marking	Scraping	Raking their hind feet through the substrate and then sometimes urinating and/or defecating on the scraped mound of material
	Urine Spraying	Spraying urine backwards onto a tree trunk or other vertical surface
	Faecal Deposition	Defecating; also called “faecal marking” in some studies
Body Rubbing	Cheek Rubbing	Rubbing their cheek on a tree trunk or other object
	Claw Marking	Raking, gouging, or gripping a tree trunk with their claws
	Rolling	Rolling back and forth on the ground; also called “vegetation flattening” in some studies
	Tail Wrapping	Wrapping their tail around a vertical tree trunk
Vocalizing	Vocalizations	Making calls or other sounds
Investigating	Olfaction	Sniffing to investigate cues and signals, noted by the clouded leopard’s nose within one head length of a scrape or other cue; also called “sniffing” in some studies
	Flehmen Response	Lifting their head and curling back their upper lip, sometimes arching its neck backwards, in order to expose their vomeronasal organ
