# Supplementary material for: Scent marking in Sunda clouded leopards (Neofelis diardi): novel observations close a key gap in understanding felid communication behaviours
Source: Sci Rep. 2016 Oct 14;6:35433. doi: 10.1038/srep35433 (PMC5064369; doi:10.1038/srep35433)
Supplement: Supplementary Information [file srep35433-s1.pdf]

Supplementary Video Captions

Scent marking in Sunda clouded leopards (*Neofelis diardi*): novel observations close a key gap in understanding felid communication behaviours

Maximilian L. Allen, Heiko U. Wittmer, Endro Setiawan, Sarah Jaffe, and Andrew J. Marshall

Video 1. Scraping behaviour

Video 2. Urine spraying behaviour

Video 3. Cheek rubbing behaviour

Video 4. Tail wrapping behaviour

Video 5. Olfaction and flehmen response behaviours

Video 6. Vocalizations
